# Identification of the Novel Small Compound Stress Response Regulators 1 and 2 That Affect Plant Abiotic Stress Signaling

**DOI:** 10.3390/biom14091177

**Published:** 2024-09-19

**Authors:** Seojung Kim, Tae-Houn Kim

**Affiliations:** 1 Department of Bio-Health Convergence, Duksung Women’s University, Seoul 01369, Republic of Korea; seojung25@gmail.com; 2 Department of Biotechnology, Duksung Women’s University, Seoul 01369, Republic of Korea

**Keywords:** chemical screening, chemical priming, small molecules, abiotic stress, plant stress-tolerant response

## Abstract

Abiotic stresses, such as drought, salinity, and extreme temperatures, limit plant growth and development, reducing crop yields. Therefore, a more comprehensive understanding of the signaling mechanisms and responses of plants to changing environmental conditions is crucial for improving sustainable agricultural productivity. Chemical screening was conducted to find novel small compounds that act as regulators of the abiotic stress signaling pathway using the ABA-inducible transgenic reporter line. Small molecules called stress response regulators (SRRs) were isolated by screening a synthetic library composed of 14,400 small compounds, affecting phenotypes such as seed germination, root growth, and gene expression in response to multiple abiotic stresses. Seeds pretreated with SRR compounds positively affected the germination rate and radicle emergence of Arabidopsis and tomato plants under abiotic stress conditions. The SRR-priming treatment enhanced the transcriptional responses of abiotic stress-responsive genes in response to subsequent salt stress. The isolation of the novel molecules SRR1 and SRR2 will provide a tool to elucidate the complex molecular networks underlying the plant stress-tolerant responses.

## 1. Introduction

Chemical genetics has provided a powerful strategy for understanding the molecular mechanisms underlying plant growth, development, stress responses, and epigenetic regulation using small molecules. Small molecules can modulate complex biological systems in rapid, reversible, conditional, and dose-dependent manners [1,2]. Small molecule-based approaches can thereby overcome the limitations of classical genetic studies, such as mutant lethality and genetic redundancy [3]. Small bioactive molecules that activate cognate targets and signaling pathways have been isolated by the high-throughput screening of diverse chemical libraries, allowing the discovery of novel regulatory components in many phytohormone signaling networks [4,5,6,7]. The plant hormone abscisic acid (ABA) is a pivotal regulator mediating various physiological processes of plant growth, development, and adaptive responses to environmental stresses [8]. Plants produce ABA in response to abiotic stresses, which initiate various adaptive stress responses by controlling the ion channel activities or stress-responsive gene expression [9,10]. Recently, many chemical compounds have been identified to examine the underlying mechanisms for abiotic stress responses in plants. For example, small compounds, such as pyrabactin, quinabactin, ABA ANTAGONIST1 (AA1), S7, and [5-(3,4-dichlorophenyl)furan-2-yl]-piperidine-1-ylmethanethione (DFPM), were isolated by screening a small molecule library to target the critical parts of the ABA signaling pathway [11,12,13,14,15]. Moreover, the structure-based modification on such chemical modulators has resulted in developing promising ABA agonists, such as cyanabactin, opabactin, and ABA mimic 1 fluorine derivative 4 (AMF4), enhancing responses compared to ABA [4,16,17].

Chemical priming has recently been used to improve plant tolerance against a range of individual or combined abiotic stresses [18]. In a process called the chemical priming of plants, exogenous treatments with certain chemical compounds can enhance a broad range of tolerance against abiotic stresses by inducing molecular and physiological defense mechanisms. For example, different chemical priming agents such as phytohormones, reactive oxygen species (ROS), vitamins, osmoprotectants, and mineral elements cause a priming state in plants. Plants under primed conditions can improve cell homeostasis, plant growth, and crop yield because of the increased resistance to multiple abiotic and biotic stresses [19,20,21,22,23,24]. Chemical priming can induce short- or long-term stress memory, making plants more tolerant of additional stress in the current or subsequent generations. In particular, plant stress memory is mediated by epigenetic mechanisms with fundamental roles in maintaining genome stability and controlling gene expression through DNA methylation and histone modifications [25,26]. For example, a positive feedback loop between HSFA2 and the H3K27me3 histone demethylase RELATIVE OF EARLY FLOWERING 6 (REF6) contributes to a transgenerational thermomemory in Arabidopsis [27].

Priming with chemical agents can be applied at different developmental stages of the plant life cycle and to various plant parts or organs [28]. Seed priming technology is a simple, low-cost, ecofriendly, and effective strategy to improve seed and crop performance. This technology has been applied successfully in many field crops, including rice, wheat, barley, maize, sorghum, and soybean [29,30]. Seed priming is a pre-sowing, controlled seed hydration treatment that allows pre-germinative metabolic activities to continue without radical emergence. Primed seeds can rapidly start and revive the seed metabolism after sowing, leading to an improved germination rate, uniformity and vigor of seedlings, a well-developed root system, early crop establishment, and increased tolerance to abiotic and biotic stresses [29,31,32,33]. Furthermore, seed priming with combinations of different priming agents effectively enhanced seed and crop performance [34,35,36]. Despite the various organic and inorganic chemicals being used as seed priming agents [30,37,38], further studies are needed to determine the optimal treatment conditions and application methods in different crop species [39,40].

This study conducted high-throughput chemical screening using the transgenic Arabidopsis expressing *pRAB18:GFP* to identify novel small compounds that regulate abiotic stress signaling. We hypothesized that isolated small compounds might activate stress response pathways, enhancing the resistance to environmental stress in plants. Small molecules called SRRs (stress response regulators) were isolated by screening the 14,400-compound Maybridge HitFinder^TM^ library, affecting stress responses, including seed germination, root growth, and gene expression in response to multiple abiotic stresses. Interestingly, seed priming with SRR chemicals improved the germination percentage and early radicle emergence of Arabidopsis and tomato seeds under abiotic stress conditions. The SRR-priming treatment affected the transcriptional levels of stress-responsive genes and expression patterns of genes in the epigenetic control process in response to subsequent salt stress. The use of the newly identified small molecules SRR1 and SRR2 may provide an efficient tool for the comprehensive understanding of stress-tolerant responses and developing priming processes toward improved stress-tolerant traits in plants.

## 2. Materials and Methods

### 2.1. Root Growth Assay

Arabidopsis seeds (Columbia–0 (Col-0)) were sterilized with 100% ethanol and sown on growth medium (0.5× Murashige and Skoog (MS), 0.05% MES, 1% sucrose, 0.8% plant agar, pH 5.8). After two days of stratification at 4 °C, plants were grown vertically for seven days at 22 °C under long-day conditions (16 h light/8 h dark). Seven-day-old seedlings were transferred to a new solid medium containing each chemical at various concentrations. The ends of each primary root of seedlings were marked and monitored to observe root growth arrest after six more days. All root growth experiments were repeated at least three times in independent experiments.

### 2.2. Chemicals

SRR1 (HTS13184SC) and SRR2 (JFD02841SC) were isolated from the screening of 14,400 small synthetic compounds in the Maybridge HitFinder^TM^ library (Thermo Fisher Scientific, Waltham, MA, USA) (http://www.maybridge.com/ (accessed on 10 January 2018)). The compound library has high structural diversity and purity levels greater than 90%, following the Lipinski guidelines for drug-likeness. The library compounds were dissolved in 100% DMSO at 10 mM.

### 2.3. Chemical Library Screening 

The homozygous T3 transgenic reporter line expressing *pRAB18:GFP* in the Col-0 background was used for chemical library screening [14]. Ten-day-old transgenic reporter lines grown in 0.5× MS liquid medium in 96-well plates were incubated for 15 h with each library compound at a final concentration of 50 μM. Eighty different library compounds were stored in the middle of a 96-well plate, and the first and last columns were used as controls without chemicals. Eight positive controls (10 μM ABA) and eight negative controls (distilled water) were located alternately on the first and last columns of the plate to minimize errors. 0.1% DMSO was used as the solvent control for chemicals, and 0.02% ethanol was used as the solvent control for ABA. After the chemical incubation, compound-induced GFP fluorescence was measured using a NIGHTSEA™ stereo microscope fluorescence adapter (NIGHTSEA, Hatfield, PA, USA) with a green filter set. The compounds that induced a significant pRAB18:GFP expression were considered hit compounds. The hit chemicals were reexamined through secondary and tertiary screenings using confocal microscopy.

### 2.4. Quantitative Real-Time PCR

Arabidopsis, tomato, and radish seedlings grown on growth media for 12 days were treated with DMSO, 10 μM ABA, 200 mM NaCl, or 50 μM SRR compounds for six hours. The total RNA was extracted using TRIsure™ (Meridian Bioscience Inc., Memphis, TN, USA) and treated with DNase I (Thermo Fisher Scientific, Waltham, MA, USA) before reverse transcription using a SensiFAST™ cDNA Synthesis Kit (Meridian Bioscience Inc., Memphis, TN, USA). The mRNA expression of the target gene was measured by RT-qPCR using SensiFAST™ SYBR^®^ Hi-ROX Kit (Meridian Bioscience Inc., Memphis, TN, USA) and normalized relative to the expression of *Clathrin* (At4g24550), *CAC* (Solyc08g006960), or *ACTIN* (Rsa1.0_00546.1) as reference genes (Table S1). Four replicates were examined for each condition.

### 2.5. RNA-Seq and Co-Expression Analysis 

Twelve-day-old Arabidopsis Col-0 seedlings treated with 0.1% DMSO, 10µM ABA, 200 mM NaCl, or 50 µM SRR chemicals for six hours were used for RNA extraction with TRIsure™ (Meridian Bioscience Inc., Memphis, TN, USA) according to the manufacturer’s instructions. Purified RNA was treated with DNase I (Thermo Fisher Scientific, Waltham, MA, USA) and evaluated for its quality by determining the RNA Integrity Number (RIN). Three biological replicates of each treatment group, with a RIN value > 7, were used to construct a cDNA library. Sequencing of the cDNA libraries was performed using an Illumina HiSeq4000 (Illumina, Inc., San Diego, CA, USA) with a 100 bp read length and a sequence depth of ~50 million uniquely mapped reads. After a quality assessment and the pre-processing of raw sequence data, the filtered reads were aligned by HISAT2 to the Arabidopsis TAIR10 genome and assembled into transcripts using StringTie. Differentially expressed genes (DEGs) between each sample were selected based on the false discovery rate (FDR) < 0.05 and |log2 fold-change (FC)| ≥ 2 and subjected to Gene Ontology (GO) functional enrichment (http://www.geneontology.org (accessed on 11 January 2019)) and Kyoto encyclopedia of genes and genomes (KEGG) pathway analysis (http://www.genome.jp/kegg (accessed on 11 January 2019)).

All interaction evidence obtained in the STRING database (version 11, https://string-db.org/ (accessed on 19 October 2022)) was used to retrieve the predicted protein–protein interaction (PPI) networks, including text mining, experiments, databases, co-expression, neighborhood, gene fusion, and co-occurrence [40]. The TAIR homologous proteins of *Arabidopsis thaliana* were mapped using the STRING system and visualized in the interaction network at a high confidence level of >0.7. The network nodes correspond to the proteins, and the edges represent the predicted functional associations.

### 2.6. Seed Priming and Germination Assay

Tomato (*Solanum lycopersicum*) and rashish (*Raphanus sativus*) seeds were pretreated with 0.01% rapidase for three hours and washed three times with distilled water. After the pretreatment, the seeds were briefly sterilized with 70% ethanol and rinsed twice with distilled water. The seeds were finally treated with 50% chlorine solution for 30 s, followed by re-washing ten times with distilled water to remove the residual disinfectant. Sterilized Arabidopsis seeds were primed by soaking in different priming solutions for 24 h at room temperature in the dark. Various chemical solutions were used as priming agents: distilled water, 1% KNO_3_, 1% NaCl, and five concentrations of the SRR chemicals (1, 10, 30, 50, and 100 μM). The seed weight to solution volume ratio (*w*/*v*) was 1:5. Distilled water-primed seeds were used as the control. All primed seeds were washed with distilled water for two minutes and re-dried to their original moisture content on filter paper before sowing. After the priming process, seeds were sown on half-strength MS medium supplemented with or without a different concentration of chemicals: 0.5 μM ABA, 150 mM NaCl, 6% PEG-6000, and 200 mM mannitol. After two days of stratification at 4 °C, the seeds were grown for seven days at 22 °C under long-day conditions (16 h light/8 h dark). The germination rate was evaluated six days after sowing, and each treatment was replicated three times with 60 seeds per replicate.

### 2.7. Transcriptional Analysis for Successive Stress Conditions

Ten-day-old seedlings were pretreated with 100 mM NaCl or each concentration of SRR compounds for 24 h. The primed seedlings were recovered in liquid growth media for 48 h and subjected to second salt stress with 200 mM NaCl for six hours. Distilled water-primed control seedlings were recovered in parallel before being treated with 200 mM NaCl. All plant tissues were harvested after the second stress treatment to determine the transcript level.

## 3. Results

### 3.1. High-Throughput Screening of Synthetic Chemical Libraries for a Regulator of Abiotic Stress Signaling

**Figure 1:**
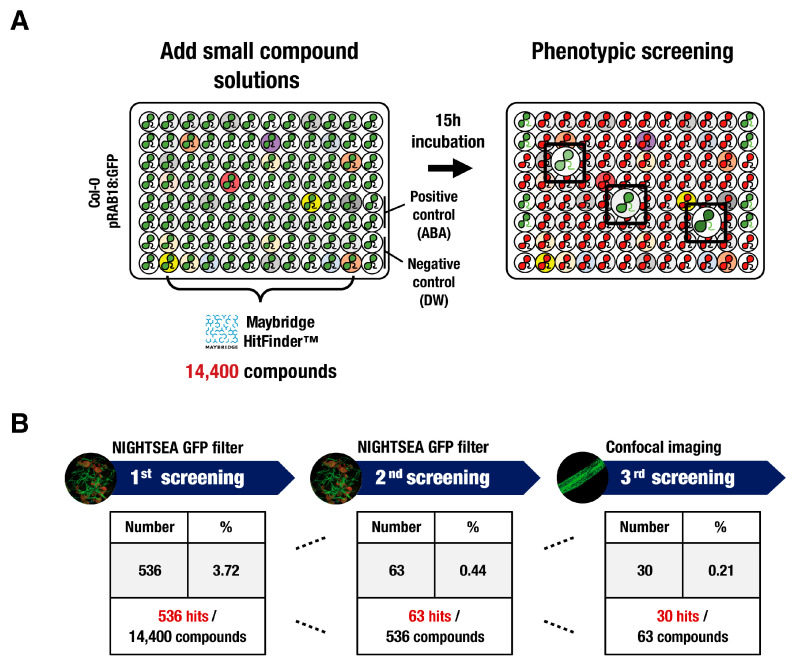
Screening of synthetic chemical libraries for a regulator of abiotic stress signaling. (**A**) Schematic representation of chemical library screening. Ten-day-old *pRAB18:GFP*-expressing seedlings grown in liquid MS medium on 96-well plates were incubated for 15 h with library compounds at a final concentration of 50 μM. After incubation, the chemical-induced GFP fluorescence signal was analyzed using a NIGHTSEA™ stereo microscope fluorescence adapter and confocal microscopy. A treatment with 10 μM ABA or distilled water was used as a control. (**B**) Summary of results for each screening. The number and the percentage of chemicals that fall into each screening are shown.

Forward chemical genetic screening was performed to identify novel small compounds that potentially regulate the abiotic stress signal transduction using the transgenic Arabidopsis seedlings expressing the ABA-responsive reporter gene *pRAB18:GFP*. Candidate molecules that induce the ABA-mediated *pRAB18:GFP* reporter expression were isolated by screening a highly diverse set of 14,400 small compounds from the Maybridge HitFinder^TM^ library (Figure 1A). Ten-day-old transgenic seedlings grown in 96-well tissue culture plates were incubated with each library compound at a concentration of 50 μM for 15 h. After the chemical incubation, chemically induced GFP fluorescence was measured using a NIGHTSEA™ stereo microscope fluorescence adapter. The compounds that induced significant *pRAB18:GFP* expression in the leaves or roots of the transgenic seedlings were regarded as hit compounds. Five hundred and thirty-six chemicals were identified in the initial screening, corresponding to a 3.72% hit rate among all compounds (Figure 1B). Secondary screening was conducted for candidates with high GFP expression intensity, which was first selected and narrowed down to 63 hit compounds. The selected hit chemicals were re-examined through tertiary screening by confocal microscopy to detect specific GFP expression in the epidermal layer of true leaves or roots. Among the screening compounds, 30 small molecules that produced a significant GFP reporter induction in epidermal cells were analyzed further.

### 3.2. Small Molecules SRR1 and SRR2 Affect the Physiology of Seed Germination and Root Growth

**Figure 2:**
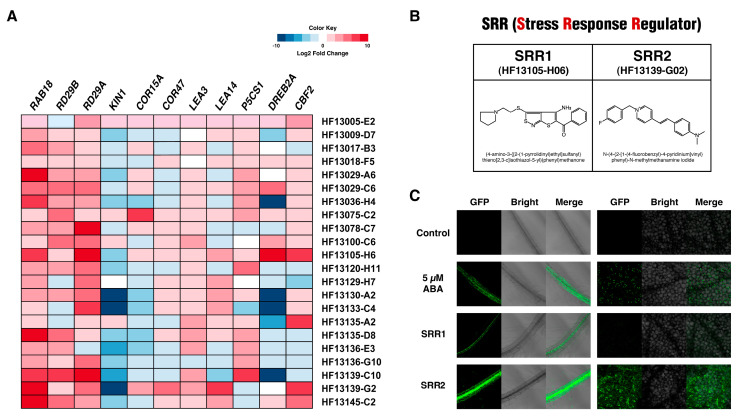
Small molecule SRRs induce the abiotic stress-inducible gene expression. (**A**) Among the 30 candidate compounds, 22 small molecules caused a significant induction of abiotic stress-responsive gene expression. The expression levels were determined by RT-qPCR analysis. The color key at the top shows a log2-fold change. (**B**) Chemical structures of the SRR1 and SRR2 compounds. (**C**) Representative images of *pRAB18:GFP* reporter expression in epidermal cells of leaves and roots after treatment with DMSO, 5 μM ABA, or 50 μM SRR compounds.

The expression of several abiotic stress-responsive genes was analyzed to determine if the candidate compounds activate the endogenous expression level of stress-responsive genes in addition to the *RAB18* reporter gene. Of the 30 candidate compounds, 22 small molecules significantly increased the expression of genes induced by multiple abiotic stress stimuli such as salt, drought, cold, and ABA (Figure 2A). These small compounds were called stress response regulators (SRRs). In particular, two small molecules were selected for further analysis, designated SRR1 (4-amino-3-{[2-(1-pyrrolidinyl)ethyl]sulfanyl}thieno [2,3-c]isothiazol-5-yl)(phenyl)methanone) and SRR2 (N-(4-{2-[1-(4-fluorobenzyl)-4-pyridinium]vinyl}phenyl)-N-methylmethanamine iodide), which significantly increased the expression levels of several abiotic stress-inducible genes (Figure 2A,B). SRR1 and SRR2 induced *RAB18* reporter gene expression to the extent induced by 5 μM ABA in epidermal cells (Figure 2C).

**Figure 3:**
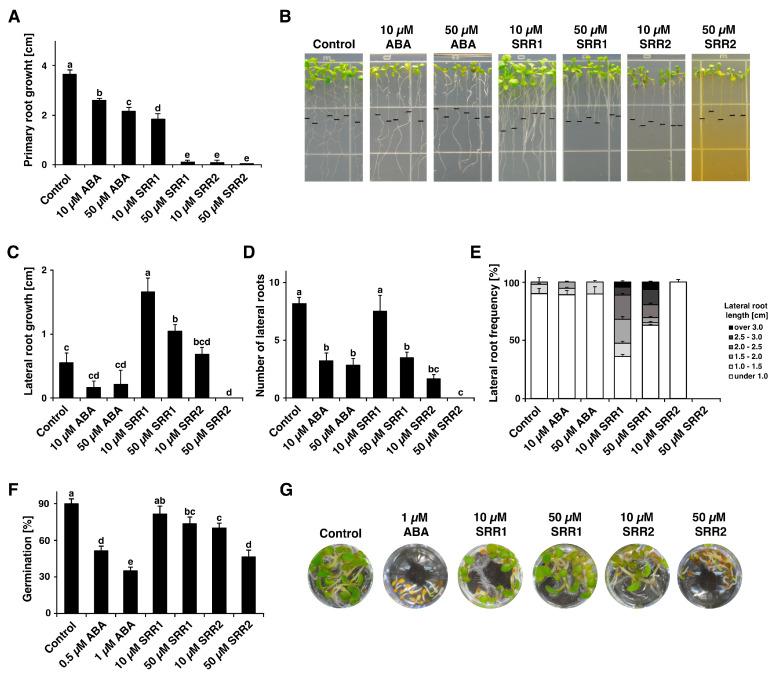
SRR1 and SRR2 affect root growth and seed germination. (**A**) SRR1 and SRR2 caused the primary root growth arrest. Seven-day-old Col-0 seedlings were treated with SRR and monitored for root growth after six days. (**B**) Representative photographs of Col-0 seedlings grown on MS media containing DMSO, ABA, SRR1, and SRR2. The black horizontal bars mark the starting point of the root tips when the plants were transferred to plates with the designated chemicals. (**C**) The lateral root length, (**D**) lateral root number, and (**E**) lateral root frequency of plants treated with the selected compounds were measured. (**F**) SRR1 and SRR2 exhibited inhibitory activities on seed germination. The bar graphs show the seed germination rate percentage after five days of sowing. (**G**) The photographs were taken seven days after sowing. DMSO treatment was used as a control. The error bars represent the SD of the mean (n = 3 with 12 plants each). Different letters represent significant differences determined by one-way ANOVA with Tukey’s post-hoc test (*p* < 0.05).

The effects of the selected SRR compounds on root growth and seed germination were analyzed. Seven-day-old seedlings grown on a standard growth medium were transferred to the plates with each concentration of the chemicals and monitored for the production of any root growth arrest phenotype for a week. Treatment with SRR1 caused a substantial decrease in primary root growth while increasing the average lateral root length compared to the DMSO or ABA treatment (Figure 3A,C,E). By contrast, the application of SRR2 severely inhibited primary root growth (Figure 3A) and reduced the number and length of lateral roots at a low concentration of 10 μM (Figure 3C,D). The germination rate was decreased by approximately 73% and 46% after 50 μM SRR1 and SRR2 treatments, respectively (Figure 3F).

### 3.3. SRR1 and SRR2 Trigger the Expression of Genes Involved in Abiotic Stress Responses

**Figure 4:**
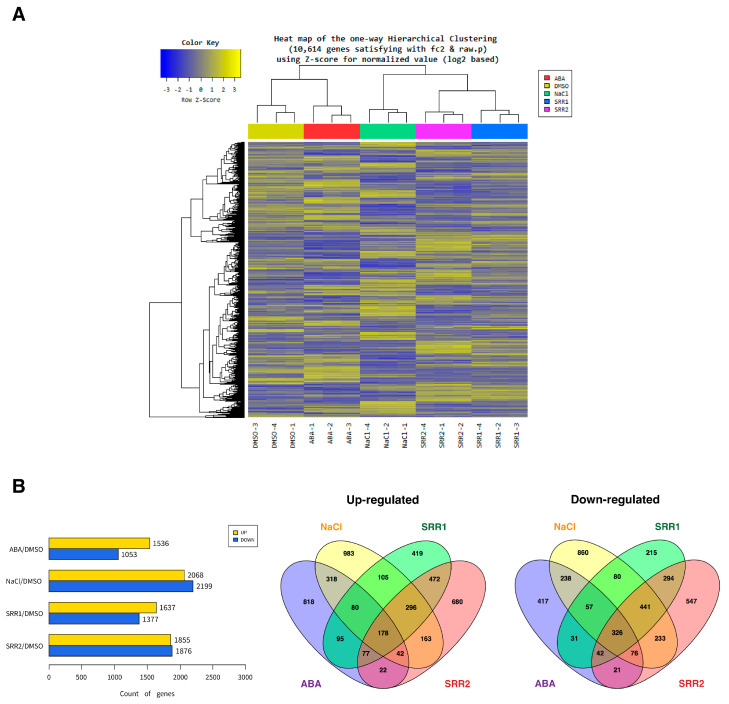
Transcriptome analysis for the differentially expressed genes (DEGs) induced by selected SRR compounds. (**A**) Cluster heatmap of DEGs under ABA, salt, and SRR chemicals treatments. Twelve-day-old seedlings were treated with DMSO, 10 µM ABA, 200 mM NaCl, and 50 µM SRR chemicals for six hours. Differentially expressed genes (DEGs) were isolated between three biological replicates of each treatment group based on the |log2 FC| ≥ 2 and adjusted for *p* < 0.05. Treatment with 0.1% DMSO was used as a control. (**B**) Venn diagrams and bar graphs of up- and downregulated DEGs in different treatment groups.

The selected chemicals SRR1 and SRR2 significantly upregulated the expression of well-characterized marker genes, including *RD29A*, *DREB2A*, *CBF2*, and *LEA14*, which are responsible for imparting enhanced tolerance to various abiotic stresses [41,42,43,44,45]. RNA-sequencing analysis investigated the large-scale gene expression profiles triggered by SRR1 and SRR2. As a result, 10,614 differentially expressed genes (DEGs) were isolated between each treatment group (Figure 4A). Among the DEGs induced by SRR compounds, the SRR1-treated group contained 3014 DEGs, of which 1637 were upregulated and 1377 were downregulated (Figure 4B). In addition, the SRR2 treatment produced 1855 upregulated DEGs and 1876 downregulated DEGs. In particular, many SRR-induced DEGs exhibited differential expression across ABA or salt treatments, suggesting that the application of SRR compounds induces responses similar to those for abiotic stresses (Figure 4B). Gene Ontology (GO) enrichment analysis for these DEGs presented that the SRR-induced DEGs were enriched significantly in the main terms responsible for controlling the plant resistance to abiotic stresses, such as the oxidation-reduction process, sequence-specific DNA binding, and integral component of the membrane (Figure S1A) [46,47,48]. In addition, the stress-responsive GO enrichments showed that SRR compounds triggered the expression of many genes involved in the response to multiple abiotic stresses (Figure S1B).

In particular, SRR compounds upregulated the transcript levels of several gene family members involved in the essential steps of the abiotic stress response and tolerance, including *HSPs* and *GSTUs* (Tables S2 and S3) [49,50,51,52,53,54,55,56,57,58,59,60]. Hence, SRR chemicals may regulate the plant responses to various abiotic stresses through the transcriptional control of the signaling components participating in multiple stress responses.

### 3.4. Selected SRR Compounds Induce the Abiotic Stress-Related Gene Expression in Crop Plants

**Figure 5:**
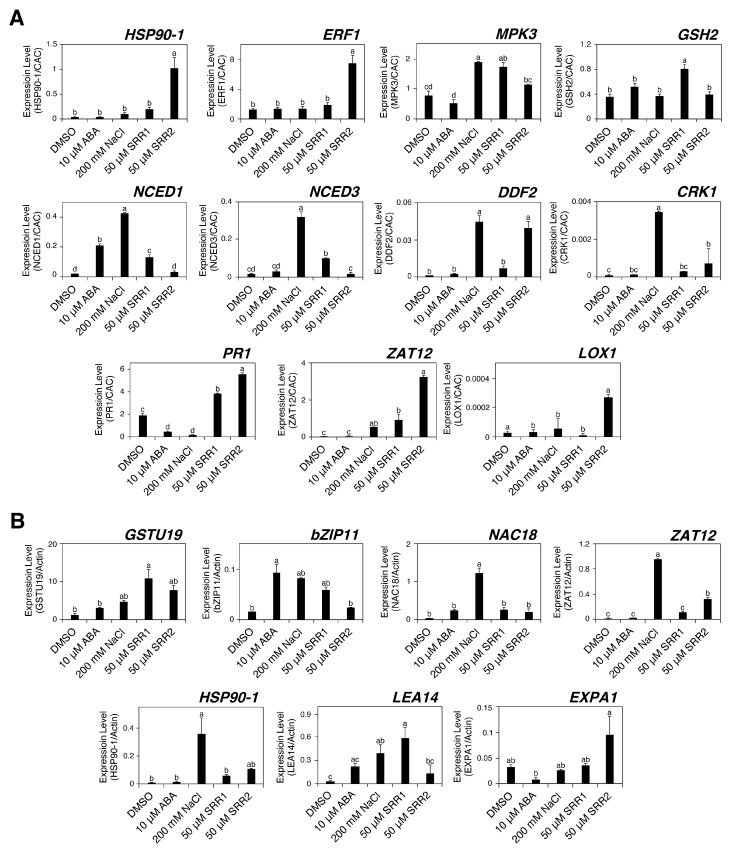
Analysis of the abiotic stress-responsive gene expression changes in crop plants by SRR1 and SRR2 treatment. The expression level of the abiotic stress-related genes in (**A**) tomato and (**B**) radish was determined by RT-qPCR analysis. Ten-day-old seedlings were treated with 10 μM ABA, 200 mM NaCl, or 50 μM SRR compounds for six hours. Treatment with DMSO was used as a control. The error bars indicate the SD of the mean (n = 3). Different letters represent significant differences determined by one-way ANOVA with Tukey’s post-hoc test (*p* < 0.05).

In Arabidopsis, SRR1 and SRR2 could influence abiotic stress-induced gene expression. This study examined whether the effects of SRR1 and SRR2 similarly activate abiotic stress signaling in crops such as tomatoes and radishes. Ten-day-old tomato and radish seedlings were treated with 10 µM ABA, 200 mM NaCl, or 50 µM SRR chemicals for six hours to determine the expression levels of the abiotic stress-responsive genes. Overall, the SRR treatments caused transcriptional changes in several genes involved in the abiotic stress responses in crop plants. The transcript levels of *MPK3*, *PR1*, and *ZAT12*, known for mediating multiple stress responses, were increased significantly by SRR1 more than by ABA or salt treatment in tomatoes (Figure 5A) [61,62,63]. The SRR1 treatment increased the expression of critical enzymes required for ABA biosynthesis, such as *NCED1* and *NCED3* [64]. The SRR2 treatment increased the expression levels of most of the examined genes compared to the control [65,66,67,68,69,70]. In addition, several genes associated with salinity or drought stress tolerance, such as *LEA14*, *GSTU19*, and *bZIP11*, were upregulated significantly by the SRR1 treatment compared to the control in radish (Figure 5B) [44,71,72]. The SRR2 treatment in radish increased the expression level of all investigated genes more than the control. These results suggest that SRR1 and SRR2 could be universal regulators of abiotic stress responses in various crops.

### 3.5. Seed Priming with SRR1 and SRR2 Affects Germination and Radicle Emergence of Arabidopsis and Tomato Seeds

**Figure 6:**
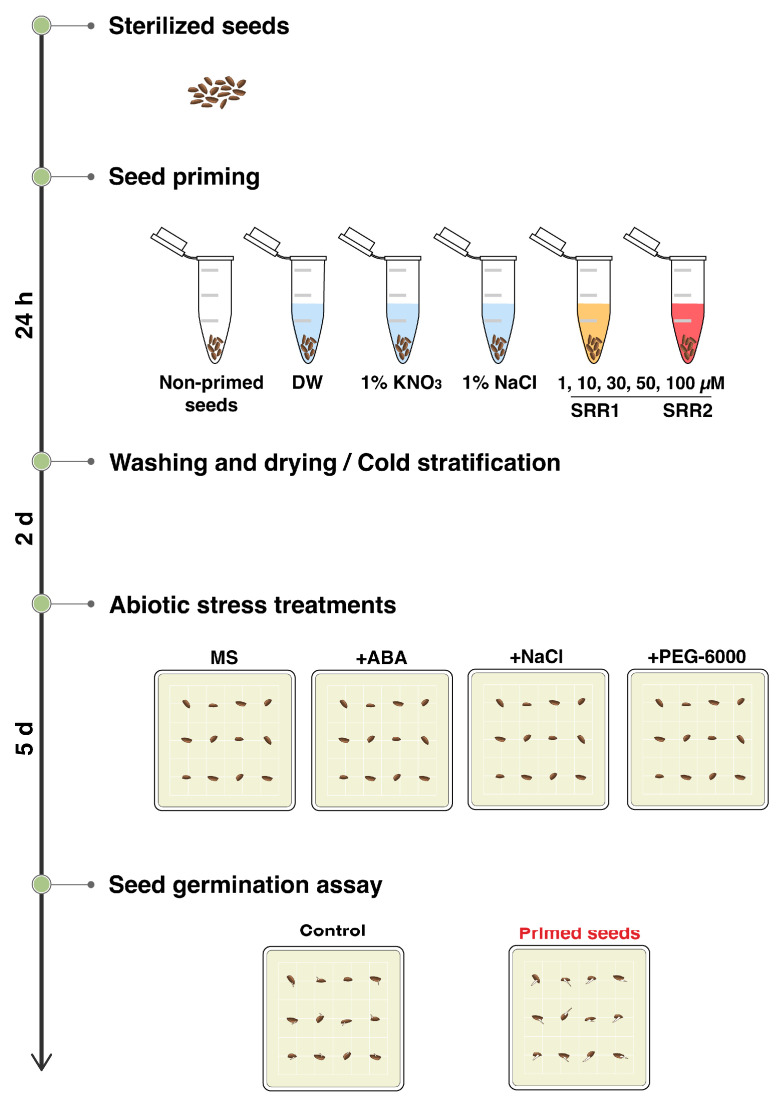
Schematic diagram of the experimental procedure for seed priming. Sterilized seeds were soaked in different priming solutions for 24 h: distilled water (DW), 1% KNO_3_, 1% NaCl, and various concentrations of SRR chemicals (1, 10, 30, 50, and 100 μM). The primed seeds were washed and re-dried to their original moisture content before sowing. After two days of stratification at 4 °C, seeds were grown on MS medium supplemented with 0.5 μM ABA, 150 mM NaCl, or 6% PEG-6000. The germination rate was measured after five days of sowing. The distilled water-primed seeds were used as the control.

**Figure 7:**
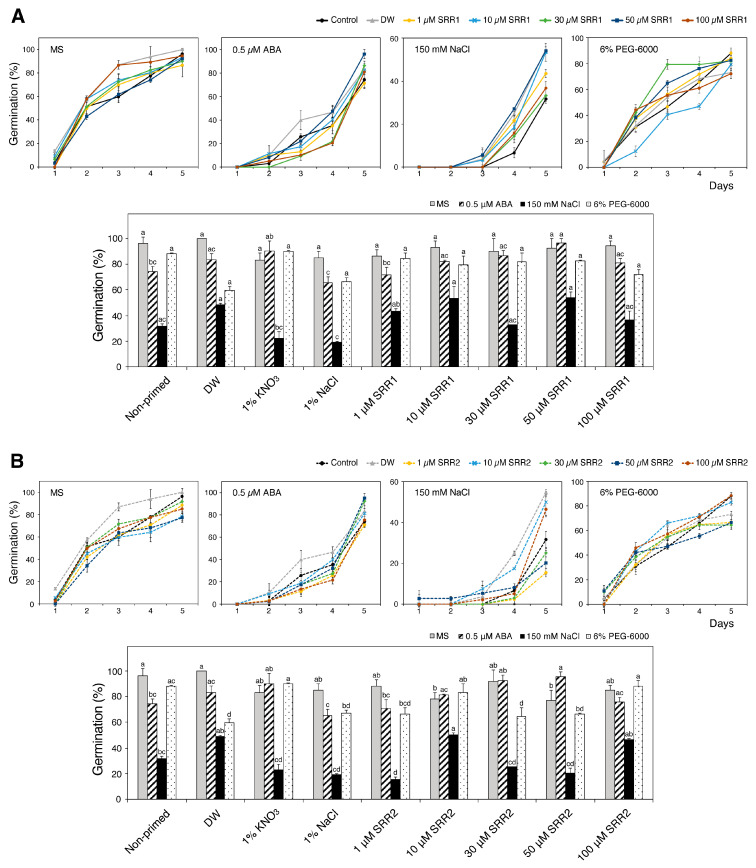
Effects of seed priming with SRR1 and SRR2 on Arabidopsis seed germination under abiotic stress conditions. Seed priming treatments with (**A**) SRR1 and (**B**) SRR2 affected Arabidopsis seed germination. Seeds were primed in different priming solutions for 24 h and grown under ABA, salt, or osmotic stress conditions. The number of germinated seeds was counted from days one to five. The bar graphs show the germination rate five days after sowing. Water-primed seeds were used as a control. The error bars indicate the SD of the mean (n = 3 with 20 plants each). Different letters represent significant differences determined by one-way ANOVA with Tukey’s post-hoc test (*p* < 0.05).

Based on reports that seed priming using chemical compounds can improve plant germination and growth, this study investigated whether seed priming with SRR1 and SRR2 positively affected the physiology of seed germination under abiotic stress conditions (Figure 6). Sterilized Arabidopsis seeds were primed by soaking in five priming agent solutions for 24 h: distilled water, 1% KNO_3_, 1% NaCl, and different concentrations of SRR chemicals. The primed seeds were grown on MS medium supplemented with 0.5 μM ABA, 150 mM NaCl, or 6% PEG-6000 and evaluated for germination analysis. As a result, seed priming with selected SRR chemicals generally enhanced the germination performance of Arabidopsis seeds in response to abiotic stresses. The primed seeds with SRR1 concentrations ranging from 10 to 50 µM gradually increased the germination percentage under ABA stress (Figure 7A). Priming with all concentrations of SRR1 resulted in a higher germination rate and faster radicle protrusion compared to the water-primed control during PEG stress. Increasing SRR2 concentrations of up to 50 μM can also promote seed germination in response to ABA stress (Figure 7B). The exogenous application of 30 μM SRR2 significantly improved the salt tolerance during seed germination. Overall, soaking in a 50 μM SRR1 solution had the most pronounced effects on improving seed germination in response to various abiotic stresses compared to other priming agents. SRR2 priming at concentrations of 50 μM was most effective in promoting seed germination under ABA stress conditions. On the other hand, the priming effects of SRR1 against salt stress were not strong compared to the control during seed germination.

### 3.6. Priming with SRR1 and SRR2 Augments the Induction of Abiotic Stress-Responsive Genes under Salt Stress

**Figure 8:**
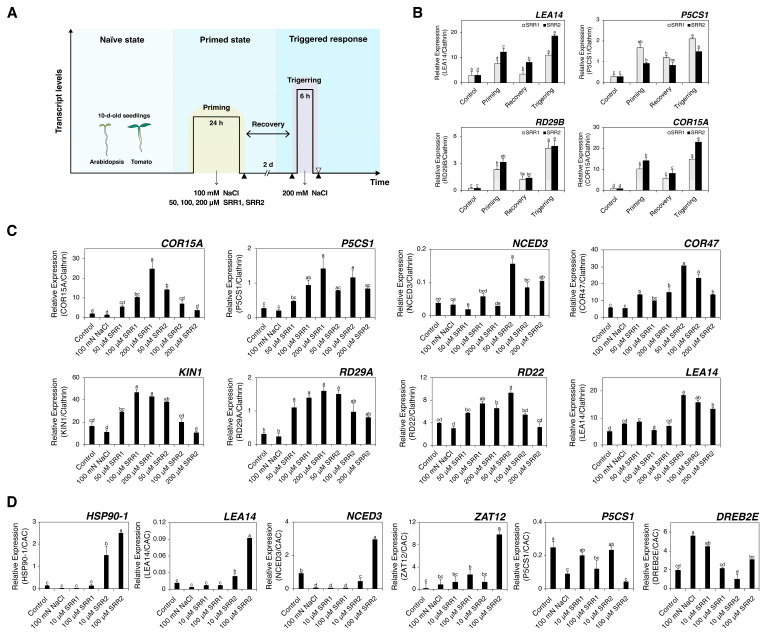
SRR-induced transcriptional amplification of the abiotic stress-responsive gene expression under subsequent salt stress. (**A**) Experimental scheme for SRR-induced transcriptional amplification assay. Ten-day-old seedlings were pretreated in distilled water, 100 mM NaCl, or each concentration of SRR compounds for 24 h (priming; primary stress). These primed seedlings were recovered in liquid growth media for 48 h and subjected to the second salt stress with 200 mM NaCl for six hours (triggering; secondary stress). Distilled water-primed seedlings were used as a control. All plant tissues were harvested immediately at the corresponding time points to determine the transcript levels (triangles). (**B**) Arabidopsis seedlings were pretreated with 50 μM SRR compounds for 24 h and recovered for two days before being treated with 200 mM NaCl. RT-qPCR determined the transcript levels after priming, recovery, and secondary stress treatments (the black triangles represent the time points). (**C**) Arabidopsis and (**D**) tomato seedlings were exposed to the treatment conditions as presented in Figure 8A. The transcript levels were determined after the second stress treatment (white triangle). As a control, distilled water-primed seedlings were recovered and used for the second stress treatment. The error bars indicate the SD of the mean (n = 3). Different letters represent significant differences determined by one-way ANOVA with Tukey’s post-hoc test (*p* < 0.05).

After confirming that the priming treatment of SRR1 and SRR2 affected the seed germination response to abiotic stress, this study examined how the SRR1 and SRR2 priming treatments affected the gene expression of abiotic stress-responsive genes in response to subsequent abiotic stress. Ten-day-old Arabidopsis and tomato seedlings were pretreated with 100 mM NaCl or each concentration of the SRR chemical solutions for 24 h. The primed seedlings recovered in growth media for two days were subjected to secondary salt stress with 200 mM NaCl for six hours (Figure 8A). All plant tissues were harvested at corresponding time points to determine the transcript levels. After the priming treatment with 50 μM SRR compounds, the transcript levels of abiotic stress-responsive genes increased to expected levels, followed by a significant decrease in the expression levels in the recovery interval (Figure 8B). The SRR-primed seedlings showed more robust transcriptional reactivation during subsequent salt stress than during the first stress exposure (Figure 8B). The priming treatment with each concentration of SRR1 caused the strong induction of stress-related maker genes, including *COR15A*, *P5CS1*, *COR47*, *RD29A*, *RD22*, and *KIN1*, compared to the control or NaCl treatment (Figure 8C) [73,74]. Similar to the SRR1-priming treatment, priming with SRR2 also increased the expression of all maker genes in subsequent salt stress (Figure 8C). Interestingly, priming treatment with SRR1 significantly increased the levels of *COR15A*, *P5CS1*, and *RD29A* expression in a dose-dependent manner (Figure 8C). On the other hand, most of the genes examined as stress-responsive genes were progressively downregulated with increasing SRR2 concentration during secondary salt stress conditions. In line with the results shown in Arabidopsis, the SRR-priming treatment in tomatoes affected the abiotic stress-induced gene expression under secondary salt stress conditions (Figure 8D). The *ZAT12* expression was upregulated slightly by SRR1-priming, whereas the levels of *P5CS1* expression were lower than that of the control. Pretreatment with SRR2 greatly enhanced the transcript levels of the *HSP90-1*, *LEA14*, *NCED3*, and *ZAT12* genes compared to the effects of the NaCl treatment. Priming with SRR1 and SRR2 might induce mechanisms to increase the transcription of abiotic stress-induced genes for successive stress conditions in Arabidopsis and tomato.

## 4. Discussion

Climate change has increased the frequency and intensity of abiotic stress combinations, which will have far-reaching impacts on plant growth and agricultural productivity [75,76]. Chemical priming is a promising tool that allows plants to activate more rapid and vigorous responses to mitigate individual or combined abiotic stresses [18]. The novel small molecules SRR1 and SRR2 were isolated by screening a 14,400-compound chemical library based on the ABA-mediated *pRAB18:GFP* reporter expression (Figure 1 and Figure 2). Phytohormones, termed plant growth regulators, are small molecules that govern all aspects of plant development and growth under optimal and stress conditions. The selected small compound SRRs can also affect the different developmental stages of plants, such as seed germination, root elongation, and lateral root formation. In particular, SRR2 caused a substantial reduction in primary root growth (Figure 3A), lateral root formation (Figure 3C,D), and seed germination (Figure 3F). The application of SRR1 consistently produced inhibitory effects on primary root growth (Figure 3A) and seed germination (Figure 3F). On the other hand, SRR1 reduced the total number of lateral roots (Figure 3D) while increasing the average lateral root length compared to the DMSO control (Figure 3C,E). In particular, the seedlings exposed to 10 μM SRR1 with less abundant but much longer lateral roots had normal green leaf phenotypes similar to that observed in the control (Figure 3B). Several root architectural traits, such as root length, branching and lateral root number, and root hair development, facilitate adaptation to multiple stress factors in different crops [77,78,79]. For example, transgenic Arabidopsis plants overexpressing *GmNAC109* exhibited an increased lateral root formation and improved tolerance to drought and salt stress [80]. Maize genotypes with fewer but longer lateral roots showed superior water capture, biomass, and yield under drought stress [81]. These results suggest that SRR compounds might have plant-growth regulatory activities, potentially improving plant tolerance against abiotic stress.

This study reported that SRR1 and SRR2 induce the transcriptional activation of genes, imparting multiple stress tolerances, which was found through comparative transcriptome profiling and co-expression network analysis (Figure 4 and Figures S1–S4). The GO annotation showed that the SRR-triggered DEGs were highly enriched into functional terms, including the response to abiotic stimulus, redox process, and membrane components (Figure S1). Many of these DEGs were enriched in the Kyoto Encyclopedia of Genes and Genomes (KEGG) pathways, particularly in hormone signal transduction, the biosynthesis of secondary metabolites, and metabolic pathways (Figure S2). Transcriptomic studies on gene enrichment and co-expression network analyses suggested that the signaling pathways of SRR molecules might integrate with complex networks involving HSPs, Ca^2+^ signals, and ROS regulatory systems to modulate abiotic stress responses and generate cross-stress tolerances.

This study assessed the priming effects of different concentrations of the SRR molecules on the germination performance of Arabidopsis seeds under abiotic stress conditions. Among the common seed priming agents, halopriming with 1% KNO_3_ increased Arabidopsis seed germination in the presence of ABA stress, while hydro-priming appeared to be more effective in improving the germination rate under ABA, NaCl, and normal conditions (Figure 7) [28,29]. Seed priming with the SRR1 compound at concentrations of 10–50 µM caused a dose-dependent increase in Arabidopsis seed germination under ABA stress (Figure 7A). The germination percentage was also increased by the SRR2-priming treatment at 30 μM and 50 μM (Figure 7B). Furthermore, the application of SRR1 and SRR2 positively affected the enhancement and acceleration of seed germination under PEG stress (Figure 7A,B). The priming process triggers the rapid imbibition of water and various metabolic changes at the cellular level that facilitate synchronized and earlier seed germination [28,82]. These metabolic changes enhance the tolerance capability of crop plants, including the synthesis of nucleic acids and proteins, cell division, ATP synthesis, the production of antioxidants, and the activation of DNA repair mechanisms [28,82]. Although most seed priming techniques cause partial seed pre-hydration and early germination events, their efficiency depends on the plant species and the selected priming technique. Furthermore, physical and chemical factors can influence priming effects, including the priming agent, duration, water potential, and temperature [25]. Therefore, the optimal priming conditions for each plant species should be evaluated to determine the potential applications of SRR compounds in the seed priming approach.

Priming can also stimulate stress tolerance mechanisms to subsequent stresses via activating stress memory [82,83]. Epigenetic regulatory mechanisms and chromatin marks have been heavily implicated in priming-dependent stress memory [84]. Recent studies revealed that seed priming improved stress tolerance by inducing epigenetic alterations and stress memory [85]. Overall, transcriptional amplification after the SRR-priming treatment involves more robust abiotic stress-related genes re-inducing under subsequent salt stress in Arabidopsis and tomato (Figure 8). Hence, the SRR-priming may induce stress memory that confers enhanced responses to additional stress exposure, indicating a possible cross-tolerance effect. The duration and inherent features of SRR-induced stress memory and stress tolerant effects must be analyzed further according to the treatment conditions. Future studies on identifying the chromatin and epigenetic marks at SRR-induced memory gene loci under abiotic stress conditions will help better understand the epigenetic-based mechanisms in abiotic stress adaptation and plant stress memory.

## 5. Conclusions

Based on the hypothesis that plant stress response regulators can be identified through chemical library screening, we have conducted a small compound library screening based on the ABA-inducible transcriptional responses. The selected SRR1 and SRR2 compounds affected the processes of Arabidopsis seed germination and root development and triggered transcriptional changes to multiple abiotic stresses. Furthermore, seed priming with SRR compounds alleviated abiotic stresses during the germination and the emergence of Arabidopsis seeds. Understanding the mechanisms for the specific regulation of stress-responsive gene expressions by SRR priming may be the next significant challenge. The isolation of novel small molecules, such as SRR1 and SRR2, and their possible application as priming agents will provide opportunities to elucidate the complex molecular mechanisms and epigenetic regulation underlying the plant stress-tolerant responses.

## Data Availability

The data involved in this study are listed in the article and its Supplementary Materials.

## Supplementary Materials

The following supporting information can be downloaded at: https://www.mdpi.com/article/10.3390/biom14091177/s1: Figure S1. Gene Ontology (GO) enrichment analysis for the SRR-induced DEGs. Figure S2. Kyoto Encyclopedia of Genes and Genomes (KEGG) enrichment analysis for the SRR-induced DEGs. Figure S3. Co-expression network analysis of *GSTU19* and comparison with the SRR-induced DEGs. Figure S4. Co-expression network analysis of *HSP17.6* and comparison with the SRR-induced DEGs. Table S1. List of primers used in this study. Table S2. The SRR-induced DEGs are involved in the glutathione metabolic process. Table S3. The SRR-induced DEGs participate in the heat-shock protein/chaperone pathway.
