# Supplementary material for: Identification of the Novel Small Compound Stress Response Regulators 1 and 2 That Affect Plant Abiotic Stress Signaling
Source: Biomolecules. 2024 Sep 19;14(9):1177. doi: 10.3390/biom14091177 (PMC11429841; doi:10.3390/biom14091177)
Supplement: Supplementary file 1 [file biomolecules-14-01177-s001.zip › biomolecules-3204476-supplementary.pdf]

Supplementary Materials

Identification of the Novel Small Compound Stress Response Regulators 1 and 2 That Affect Plant Abiotic Stress Signaling

Seojung Kim [1] and Tae-Houn Kim [1,2,*]

[1] Department of Bio-Health Convergence, Duksung Women's University, Seoul 01369, Republic of Korea; seojung25@gmail.com

[2] Department of Biotechnology, Duksung Women's University, Seoul 01369, Republic of Korea

* Correspondence: thkim@duksung.ac.kr; Tel.: +82-2-901-8358

A

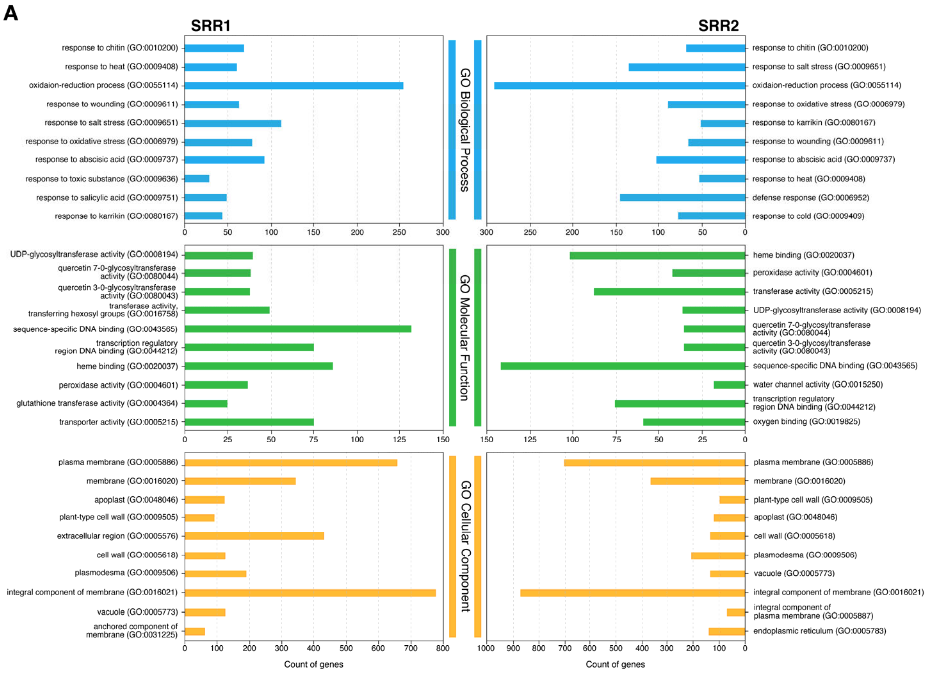

B

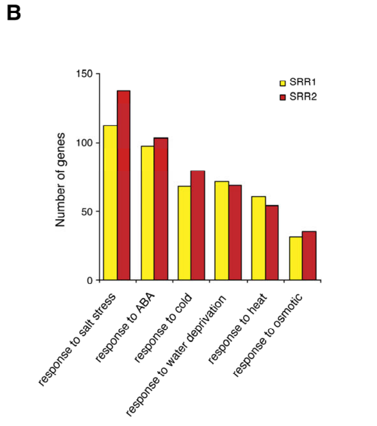

Fuctional category	SRR1	SRR2
response to salt stress	112	137
response to ABA	105	119
response to cold	68	79
response to water deprivation	71	69
response to heat	60	54
response to osmotic	31	35

Figure S1. Gene Ontology (GO) enrichment analysis for the SRR-induced DEGs. (**A**) GO enrichment analysis was performed on the differentially expressed genes induced by SRR1 and SRR2 treatments. The GO annotation represents gene sets derived from biological processes, cellular components, and molecular functions. (**B**) SRR-induced DEGs were enriched in GO categories related to abiotic stress response.

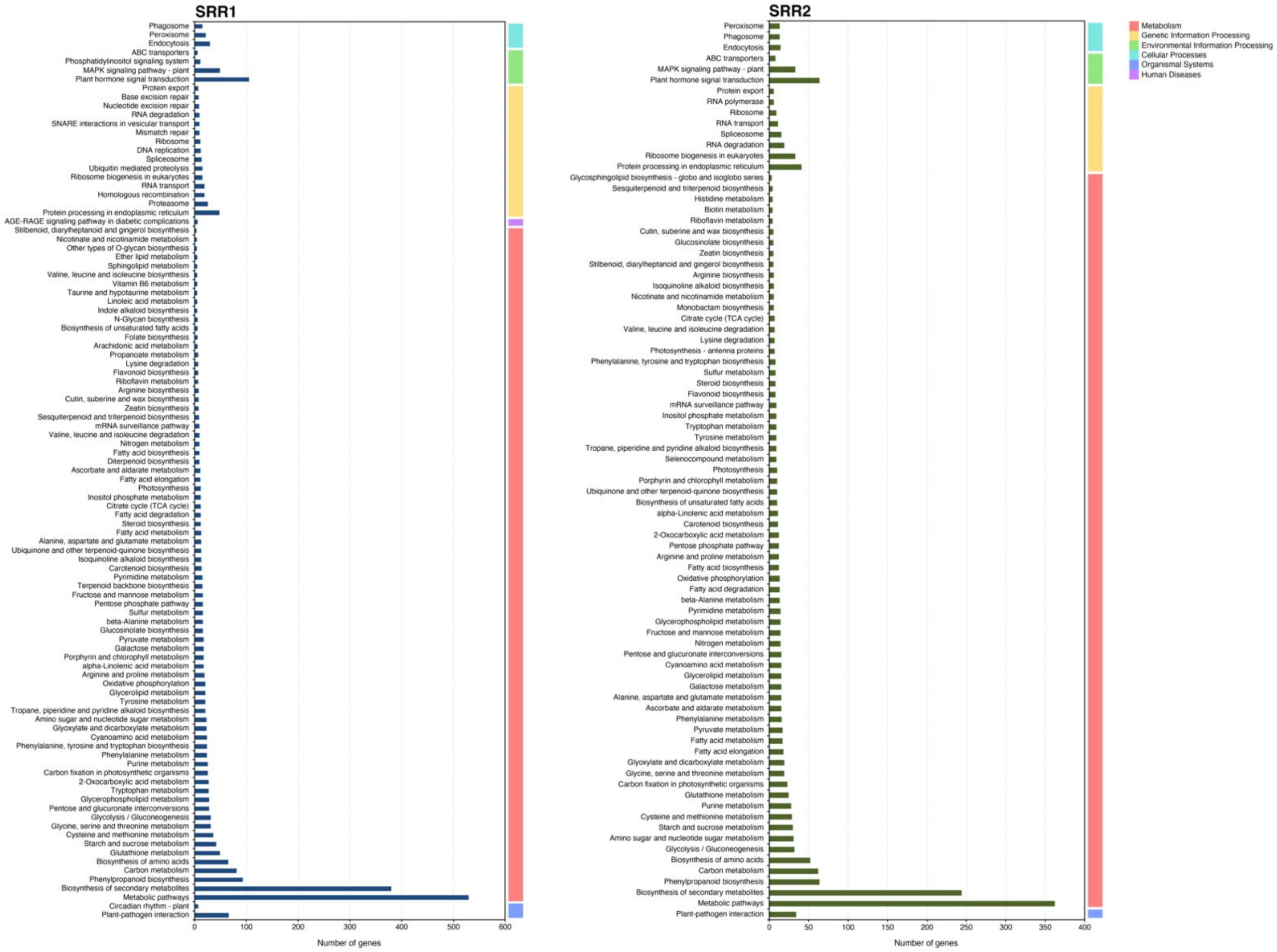

Figure S2. Kyoto Encyclopedia of Genes and Genomes (KEGG) enrichment analysis for the SRR-induced DEGs. KEGG databases annotated the SRR-induced DEGs. The vertical axis represents the name of the KEGG pathway, and the horizontal axis represents the number of DEGs annotated to the pathway. Different colors represent different KEGG pathway categories, including *metabolism*, genetic information processing, environmental information processing, cellular processes, organismal systems, and human diseases.

A

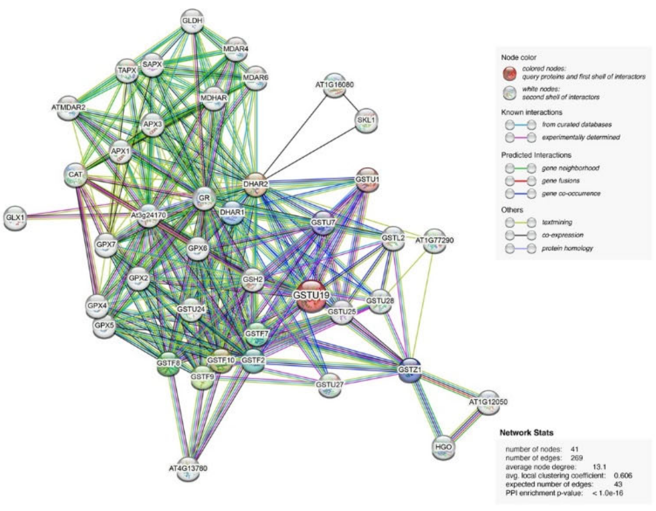

B

SRR1-induced DEGs

Gene ID	Gene Symbol	Description	Log2-fold change
AT1G17180	GSTU25	glutathione S-transferase TAU 25	251.26936
AT4G31870	GPX7	glutathione peroxidase 7	142.90080
AT1G17170	GSTU24	glutathione S-transferase TAU 24	135.02137
AT2G29420	GSTU7	glutathione S-transferase tau 7	38.99891
AT1G78380	GSTU19	glutathione S-transferase TAU 19	32.75840
AT2G29490	GSTU1	glutathione S-transferase TAU 1	27.05073
AT2G47730	GSTF8	glutathione S-transferase phi 8	17.33467
AT1G02920	GSTF7	glutathione S-transferase 7	8.38711
AT1G75270	DHAR2	dehydroascorbate reductase 2	7.89287
AT3G24170	GR1	glutathione-disulfide reductase	4.43226
AT4G11600	GPX6	glutathione peroxidase 6	3.72703
AT2G48150	GPX4	glutathione peroxidase 4	3.59230
AT4G02520	GSTF2	glutathione S-transferase PHI 2	3.55800
AT5G27380	GSH2	glutathione synthetase 2	3.34649
AT2G02390	GSTZ1	glutathione S-transferase zeta 1	3.24728
AT4G08390	SAPX	stromal ascorbate peroxidase	2.40049
AT5G03630	ATMDAR2	pyridine nucleotide-disulphide oxidoreductase family protein	2.35760
AT4G35090	CAT2	catalase 2	-2.71591
AT3G43800	GSTU27	glutathione S-transferase tau 27	-4.69062
AT1G53680	GSTU28	glutathione S-transferase TAU 28	-7.74145

SRR2-induced DEGs

Gene ID	Gene Symbol	Description	Log2-fold change
AT1G17170	GSTU24	glutathione S-transferase TAU 24	70.73179
AT2G29490	GSTU1	glutathione S-transferase TAU 1	19.97421
AT1G78380	GSTU19	glutathione S-transferase TAU 19	9.36131
AT2G29420	GSTU7	glutathione S-transferase tau 7	6.17239
AT1G02920	GSTF7	glutathione S-transferase 7	3.78893
AT4G02520	GSTF2	glutathione S-transferase PHI 2	3.69339
AT5G27380	GSH2	glutathione synthetase 2	3.28637
AT2G47730	GSTF8	glutathione S-transferase phi 8	3.21896
AT1G75270	DHAR2	dehydroascorbate reductase 2	2.21749
AT4G31870	GPX7	glutathione peroxidase 7	2.04805
AT3G24170	GR1	glutathione-disulfide reductase	2.03944
AT1G63940	MDAR6	monodehydroascorbate reductase 6	-2.33079
AT1G07890	APX1	ascorbate peroxidase 1	-2.67635
AT3G27820	MDAR4	monodehydroascorbate reductase 4	-3.23833
AT3G43800	GSTU27	glutathione S-transferase tau 27	-4.87059

Figure S3. Co-expression network analysis of *GSTU19* and comparison with the SRR-induced DEGs. (**A**) The protein–protein interaction (PPI) network of *GSTU19* was constructed using the STRING database at a high confidence level (interaction score >0.7). The PPI network is comprised of 41 nodes (proteins) and 368 edges (predicted functional associations) with PPI enrichment *p*-value <1.0e-16. (**B**) Among the 41 co-expressed genes, 20 and 15 DEGs were consistently found in the SRR1- and SRR2-treated groups, respectively.

A

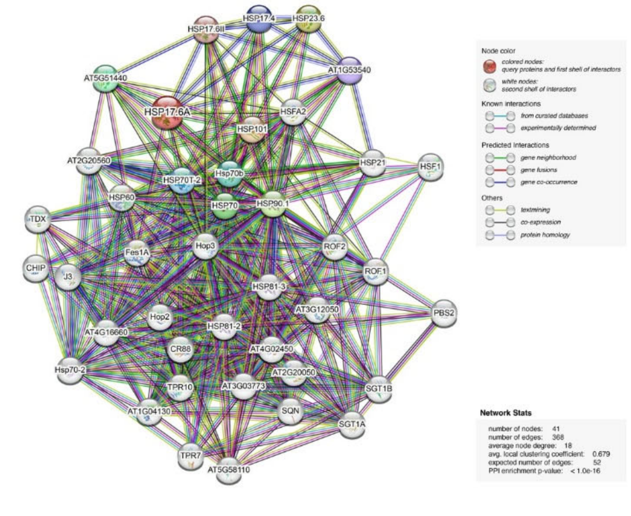

B

SRR1-induced DEGs

Gene ID	Gene Symbol	Description	Log2-fold change
AT4G25200	HSP23.6-MITO	mitochondrion-localized small heat shock protein 23.6	843.53600
AT1G53540	F22G10.20	HSP20-like chaperones superfamily protein	372.49724
AT3G46230	HSP17.4	heat shock protein 17.4	182.85866
AT5G12030	HSP17.6A	heat shock protein 17.6A	152.14764
AT5G12020	HSP17.6II	17.6 kDa class II heat shock protein	94.78191
AT5G52640	HSP90.1	heat shock protein 90.1	39.04474
AT3G12580	HSP70	heat shock protein 70	36.37363
AT5G51440	MFG13.15	HSP20-like chaperones superfamily protein	32.44529
AT2G26150	HSFA2	heat shock transcription factor A2	32.02072
AT3G09350	Fes1A	Fes1A	25.24936
AT5G02490	T22P11.80	Heat shock protein 70 family protein	22.04190
AT1G74310	HSP101	heat shock protein 101	20.97070
AT4G12400	T1P17.2	stress-inducible protein, putative	19.29669
AT5G48570	ROF2	FKBP-type peptidyl-prolyl cis-trans isomerase family protein	14.60892
AT2G20560	T13C7.15	DNAJ heat shock family protein	13.88027
AT1G16030	Hsp70b	heat shock protein 70B	12.29176
AT2G32120	HSP70T-2	heat-shock protein 70T-2	7.65882
AT3G25230	ROF1	rotamase FKBP 1	4.96900
AT5G56030	HSP81-2	heat shock protein 81-2	4.74101
AT1G62740	F23N19.10	stress-inducible protein, putative	3.41646
AT5G56010	HSP81-3	heat shock protein 81-3	3.16225
AT3G07370	CHIP	carboxyl terminus of HSC70-interacting protein	2.60510
AT3G04710	F7O18.18	ankyrin repeat family protein	2.53460
AT3G44110	ATJ3	DNAJ homologue 3	2.53045
AT4G23570	SGT1A	phosphatase-related	2.47782
AT3G12050	MEC18.18	Aha1 domain-containing protein	2.25698
AT2G15790	SQN	peptidyl-prolyl cis-trans isomerase / cyclophilin-40 (CYP40)	-2.05245

SRR2-induced DEGs

Gene ID	Gene Symbol	Description	Log2-fold change
AT4G25200	HSP23.6-MITO	mitochondrion-localized small heat shock protein 23.6	40.29462
AT5G12030	HSP17.6A	heat shock protein 17.6A	20.44931
AT1G53540	F22G10.20	HSP20-like chaperones superfamily protein	20.15115
AT3G46230	HSP17.4	heat shock protein 17.4	20.07081
AT5G51440	MFG13.15	HSP20-like chaperones superfamily protein	15.58875
AT5G12020	HSP17.6II	17.6 kDa class II heat shock protein	14.54755
AT1G16030	Hsp70b	heat shock protein 70B	13.63135
AT2G32120	HSP70T-2	heat-shock protein 70T-2	2.76511
AT1G74310	HSP101	heat shock protein 101	2.70684
AT5G52640	HSP90.1	heat shock protein 90.1	2.54773
AT1G62740	F23N19.10	stress-inducible protein, putative	2.02293
AT5G56010	HSP81-3	heat shock protein 81-3	-2.92479
AT5G02490	T22P11.80	Heat shock protein 70 family protein	-4.39242

Figure S4. Co-expression network analysis of HSP17.6 and comparison with the SRR-induced DEGs. (**A**) The protein–protein interaction (PPI) network of HSP17.6 was constructed using the STRING database at a high confidence level (interaction score >0.7). The PPI network is comprised of 41 nodes (proteins) and 368 edges (predicted functional associations) with PPI enrichment p-value <1.0e-16. (**B**) Among the 41 co-expressed genes, 27 and 13 DEGs were consistently found in the SRR1- and SRR2-treated groups, respectively.

Table S1. Primers used in this study.

Plants	Gene ID	Name	Sequence (5'-3')
Arabidopsis	At5g66400	RAB18-F	TTCACCGCTCCGGATCTGGAT
		RAB18-R	GCTCATTACACACTCATGTAAGTA
	At5g52310	RD29A-F	CTGAAGTGATCGATGCACCAG
		RD29A-R	GACACGACAGGAAACACCTTTG
	At5g52300	RD29B-F	TGGGAAGAGACTTACCGACGGAACTCATG
		RD29B-R	CGGAGAGAGGTAGCTTTGTCATCACCGTTG
	At5g25610	RD22-F	GGAGTGTGAAGCCAGAAAAG
		RD22-R	TGTGGCAGTAGAACACCGCG
	At3g14440	NCED3-F	GCCAGAAACAGATGAAGTCGTCGT
		NCED3-R	CGAATTTGGTTTTACGGCCGAGCA
	At2g42540	COR15A-F	ATGGCTTCTTCTTTCCACAGCGGAGCCAAG
		COR15A-R	TTAGGTAAGACCCTACTTTGTGGCATCCTT
	At1g20440	COR47-F	CCGTCGTAGGCTCCACCGGATG
		COR47-R	CCACTACCATCCCGGTACCAGTG
	At2g39800	P5CS1-F	TTCTTCTGAGCGATGTTGAAGGTC
		P5CS1-R	CAGCAACTGCCATGTCACGAGCAT
	At5g15960	KIN1-F	GACCAACAAGAATGCCTTCCA
		KIN1-R	GTTCAGGCCGGTCTTGTCCTTC
	At5g05410	DREB2A-F	CAGAGAGCCTAATCGAGGTAG
		DREB2A-R	CAGTACACACCTCAGACTGAC
	At4g25470	CBF2-F	GACCATGAGCATCCGTCGTCA
		CBF2-R	GCTCGGGACTTTCCAAACCGCTG
	At1g02820	LEA3-F	GCTCGTAGCTCAGCCGGGTCA
		LEA3-R	CGGCTCCGAAAAGCTCTCGAAC
	At1g01470	LEA14-F	GTCCCAGTCCACACCAACGTCTC
		LEA14-R	CGATTCCGATCTGTGAGATCAG
	At1g17730	STZ-F	TTGCTCGACTCGATACCCAG
		STZ-R	GTTGACTTCTCCTTCTGGAGT
	At3g56400	WRKY70-F	GTTTGAAGATTCCGGCGATAG
		WRKY70-R	CTCAACCTTCTGGACTTGCT
	At3g10010	DML2-F	ATTGAGGAACCAGCATCACC
		DML2-R	GCTCTGTGCGTAGCTTTTCC
	At4g34060	DML3-F	AGGGGCCTTTCCACTTAATG
		DML3-R	GCTCTTTTGGATCTCGTTGC
	At2g36490	ROS1-F	TCTCCTGCAACAGCATCA
		ROS1-R	CATGATCCGCAAACACCT
	At3g22680	RDM1-F	ATACATCTCTGCTCTTCTCAGC
		RDM1-R	CAATGACAATGGAACTACGACC
	At5g14620	DRM2-F	GATGAAACACACTTCTCCACAC
		DRM2-R	ACATCAAAGGTGTAGGAGAAGG
	At1g69770	CMT3-F	GCTAGTAAGCATAAGGAGAG
		CMT3-R	CCTTCACGTTGTAACCCATCTTCT
	At2g31650	ATX1-F	CCCAATTGCTATTCTCGAGTCATCA
		ATX1-R	TTTGCATGTTGTTCTTCAGCTTCTG
	At5g66750	DDM1-F	CAAGGCTGGAAGGGAAAG
		DDM1-R	TCGTCCATTGGGAGAAGA
	At2g34880	JMJ15-F	CCTTTGGGTTTTGTGGAGTG
		JMJ15-R	CACCAATGTCTGGCCTCTTT
	At5g49160	MET1-F	CGACAATCATAATCCGCCAATT
		MET1-R	CTGATGTTGAAGATCGTCCAAC
	At1g63020	NRPD1-F	CCAATCAGCCTTCTTGAAACT
		NRPD1-R	CTTGATCTCAAGTGACCCGT
	At3g23780	NRPD2-F	TCAAGATGGGGAAACGAAAG
		NRPD2-R	TGTGGACAAGTCGCTGGTAG

Tomato	Solyc03g007890.3.1	HSP90-1-F	TCGTAGCGGAGAAGGTTATTG
		HSP90-1-R	CAGAGTGCTTCTTTACGAGGT
	Solyc08g043170.2.1	P5CS-F	TCTTTACAGTGGTCCTCCCC
		P5CS-R	TATACGTTCCCCATGCAGCA
	Solyc06g050520.3.1	DREB1-F	GTTGGGTACATTTGGTACAGC
		DREB1-R	CCAGAAGCAACGGTGCAATC
	Solyc01g095140.3.1	LEA14-F	TCGTACACTCTCAAATGCTCC
		LEA14-R	CTCCGATGACTGGAAGATCA
	Solyc05g051200.1.1	ERF1-F	CAACAACAACAACAACAACATCA
		ERF1-R	TCCATTACGCGTTGAATCCC
	Solyc06g005170.2.1	MPK3-F	TGGAATCGTCTGCTCTGTGT
		MPK3-R	GGCGGAGGAATCACATCTCT
	Solyc01g098610.2.1	GSH2-F	CATTTCCAGGGCTAAGTTGTC
		GSH2-R	CTGGTCATACATATTGCGCTC
	Solyc07g056570.1.1	NCED1-F	TCGTGGGCTCTTCGGACTTG
		NCED1-R	CTGGGTCGAGCTTTGGGTGA
	Solyc07g056570.1.1	NCED3-F	GTATGGTTCACGCCGTTCAA
		NCED3-R	GCCTTGCAATTCCAGAGTGA
	Solyc03g026270.1.1	DDF2-F	GCCGAAATCTTCCGACCTTT
		DDF2-R	AGCTTCCACATGATCTCCCA
	Solyc03g112730.2.1	CRK1-F	TGTATTCCTGCTCCTGGTGG
		CRK1-R	CTCCTGCAGCAAAATCCCTC
	Solyc05g054655.1.1	ZAT12-F	ATCGTTTCAAGCACTAGGTGG
		ZAT12-R	GCTTAGGCGGTGAAATCGGC
	Solyc01g099190.2.1	LOX1-F	TGTCTTTGGGTGGAATTGTGG
		LOX1-R	GGATTGCTCAGTTTCCCTTCC
	Solyc01g106630.2.1	PR1-F	AACGCTCACAATGCAGCTCGT
		PR1-R	AAGGTCCACCAGAGTGTTGC
	Solyc08g006960.2.1	CAC-F	CCTCCGTTGTGATGTAACTGG
		CAC-R	ATTGGTGGAAAGTAACATCATCG
Radish	Rsa1.0_01115.1	GSTU19-F	ACTGAACATAGATAGGAACC
		GSTU19-R	GTGGACATTGGATTGATTG
	Rsa1.0_13019.1	HSP90-1-F	TTGGAGGAGAGGAGACTCAAG
		HSP90-1-R	CGTGAGAGACCTCCTTGATTT
	Rsa1.0_00048.1	bZIP11-F	TGTTCCTGTTCTGTCTTG
		bZIP11-R	CTCGCTGTTCTCTTTCTT
	Rsa1.0_00305.1	NAC18-F	GCCTTGACTATGGGTTTA
		NAC18-R	GTAACACTGAACATCGTAAC
	Rsa1.0_03900.1	ZAT12-F	CGGCGAACTGTCTGATGCTC
		ZAT12-R	GGACAAGGATGCGAAGACGA
	Rsa1.0_02288.1	LEA14-F	AAAGACGTGAACCGTAACTCC
		LEA14-R	GCACCACCATCGGAACATCAA
	Rsa1.0_00170.1	EXPA1-F	TCATTACTCCGACAATAACTA
		EXPA1-R	CATCTCTCTATCTTTCCTCTT
	Rsa1.0_00546.1	ACTIN-F	GCATCACACTTTCTACAAC
		ACTIN-R	CCTGGATAGCAACATACAT

Table S2. The SRR-induced DEGs are involved in the glutathione metabolic process.

Gene ID	Gene Symbol	Description	SRR1/DMSO

			Log2 Fold change
AT1G69930	GSTU11	glutathione S-transferase TAU 11	**424.535667**
AT2G29470	GSTU3	glutathione S-transferase tau 3	**408.979001**
AT1G17180	GSTU25	glutathione S-transferase TAU 25	**251.269361**
AT4G31870	GPX7	glutathione peroxidase 7	**142.900799**
AT1G17170	GSTU24	glutathione S-transferase TAU 24	**135.021374**
AT4G39650	GGT2	gamma-glutamyl transpeptidase 2	**80.819200**
AT2G29460	GSTU4	glutathione S-transferase tau 4	**74.304963**
AT2G29420	GSTU7	glutathione S-transferase tau 7	**38.998907**
AT2G29480	GSTU2	glutathione S-transferase tau 2	**36.227001**
AT1G78380	GSTU19	glutathione S-transferase TAU 19	**32.758402**
AT2G29490	GSTU1	glutathione S-transferase TAU 1	**27.050733**
AT1G10370	ERD9	glutathione S-transferase family protein	**19.652049**
AT2G47730	GSTF8	glutathione S-transferase phi 8	**17.334668**
AT1G69920	GSTU12	glutathione S-transferase TAU 12	**14.426405**
AT1G02930	GSTF6	glutathione S-transferase 6 (AT1G02930.1; AT1G02930.2)	**9.815181**
AT1G02920	GSTF7	glutathione S-transferase 7	**8.387109**
AT2G29450	GSTU5	glutathione S-transferase tau 5	**8.087980**
AT3G09270	GSTU8	glutathione S-transferase TAU 8	**7.983780**
AT2G29440	GSTU6	glutathione S-transferase tau 6	**7.957311**
AT1G75270	DHAR2	dehydroascorbate reductase 2	**7.892875**
AT5G02780	GSTL1	glutathione transferase lambda 1 (AT5G02780.1; AT5G02780.2)	**7.661567**
AT4G39640	GGT1	gamma-glutamyl transpeptidase 1 (AT4G39640.1; AT4G39640.2)	**4.590794**
AT2G02930	GSTF3	glutathione S-transferase F3	**4.435592**
AT3G24170	GR1	glutathione-disulfide reductase (AT3G24170.1; AT3G24170.2; AT3G24170.3)	**4.432260**
AT4G23100	GSH1	glutamate-cysteine ligase (AT4G23100.1; AT4G23100.2; AT4G23100.3)	**3.894519**
AT4G11600	GPX6	glutathione peroxidase 6	**3.727033**
AT2G48150	GPX4	glutathione peroxidase 4	**3.592303**
AT4G02520	GSTF2	glutathione S-transferase PHI 2	**3.558003**
AT1G74590	GSTU10	glutathione S-transferase TAU 10	**3.358510**

AT5G27380	GSH2	glutathione synthetase 2	3.346488
AT5G53120	SPDS3	spermidine synthase 3 (AT5G53120.1; AT5G53120.2; AT5G53120.3; AT5G53120.4; AT5G53120.5; AT5G53120.6)	3.137687
AT1G59700	GSTU16	glutathione S-transferase TAU 16	2.587463
AT3G02360	F11A12.5	6-phosphogluconate dehydrogenase family protein (AT3G02360.1; AT3G02360.2)	2.563958
AT4G08390	SAPX	stromal ascorbate peroxidase (AT4G08390.1; AT4G08390.2; AT4G08390.3; AT4G08390.4)	2.400487
AT1G65820	F1E22.17	microsomal glutathione s-transferase, putative (AT1G65820.1; AT1G65820.2; AT1G65820.3)	2.319599
AT2G24200	F27D4.11	cytosol aminopeptidase family protein (AT2G24200.1; AT2G24200.2; AT2G24200.3)	2.296347
AT5G40760	G6PD6	glucose-6-phosphate dehydrogenase 6	2.268034
AT5G13110	G6PD2	glucose-6-phosphate dehydrogenase 2	2.036021
AT2G21790	RNR1	ribonucleotide reductase 1	-2.110742
AT1G78370	GSTU20	glutathione S-transferase TAU 20	-2.158645
AT5G35790	G6PD1	glucose-6-phosphate dehydrogenase 1	-2.232589
AT5G62480	GSTU9	glutathione S-transferase tau 9 (AT5G62480.1; AT5G62480.2)	-4.397051
AT1G17190	GSTU26	glutathione S-transferase tau 26	-4.406170
AT3G43800	GSTU27	glutathione S-transferase tau 27	-4.690618
AT1G53680	GSTU28	glutathione S-transferase TAU 28	-7.741454
AT1G27140	GSTU14	glutathione S-transferase tau 14	-15.218758
AT1G49860	GSTF14	glutathione S-transferase (class phi) 14	-41.205329
AT1G78360	GSTU21	glutathione S-transferase TAU 21	-52.003615
AT3G03190	GSTF11	glutathione S-transferase F11	-183.155768
AT1G17170	GSTU24	glutathione S-transferase TAU 24	70.731789
AT2G29470	GSTU3	glutathione S-transferase tau 3	59.033526
AT2G29460	GSTU4	glutathione S-transferase tau 4	38.834919
AT5G62480	GSTU9	glutathione S-transferase tau 9 (AT5G62480.1; AT5G62480.2)	28.376757
AT2G29490	GSTU1	glutathione S-transferase TAU 1	19.974205
AT1G78380	GSTU19	glutathione S-transferase TAU 19	9.361306
AT2G02930	GSTF3	glutathione S-transferase F3	7.273087
AT5G02780	GSTL1	glutathione transferase lambda 1 (AT5G02780.1; AT5G02780.2)	6.888407
AT2G29420	GSTU7	glutathione S-transferase tau 7	6.172391
AT2G29480	GSTU2	glutathione S-transferase tau 2	6.058092

AT1G69930	GSTU11	glutathione S-transferase TAU 11	**5.329622**
AT1G69920	GSTU12	glutathione S-transferase TAU 12	**5.142543**
AT2G29440	GSTU6	glutathione S-transferase tau 6	**5.087929**
AT3G23580	RNR2A	ribonucleotide reductase 2A	**4.291878**
AT1G02920	GSTF7	glutathione S-transferase 7	**3.788925**
AT4G02520	GSTF2	glutathione S-transferase PHI 2	**3.693389**
AT2G29450	GSTU5	glutathione S-transferase tau 5	**3.349562**
AT5G27380	GSH2	glutathione synthetase 2	**3.286366**
AT2G47730	GSTF8	glutathione S-transferase phi 8	**3.218959**
AT1G75270	DHAR2	dehydroascorbate reductase 2	**2.217489**
AT4G31870	GPX7	glutathione peroxidase 7	**2.048045**
AT3G24170	GR1	glutathione-disulfide reductase (AT3G24170.1; AT3G24170.2; AT3G24170.3)	**2.039441**
AT1G07890	APX1	ascorbate peroxidase 1 (AT1G07890.1; AT1G07890.2; AT1G07890.3; AT1G07890.4; AT1G07890.5; AT1G07890.6; AT1G07890.7; AT1G07890.8)	**-2.676346**
AT3G43800	GSTU27	glutathione S-transferase tau 27	**-4.870589**
AT1G49860	GSTF14	glutathione S-transferase (class phi) 14	**-7.440765**

Table S3. The SRR-induced DEGs participate in the heat-shock protein/chaperone pathway.

Gene ID	Gene Symbol	Description	SRR1/DMSO

			Log2 Fold change
AT1G52560	F6D8.22	HSP20-like chaperones superfamily protein (AT1G52560.1; AT1G52560.2)	**5065.084771**
AT4G25200	HSP23.6-MITO	mitochondrion-localized small heat shock protein 23.6	**843.535995**
AT1G18830	F6A14.8	transducin/WD40 repeat-like superfamily protein	**834.667195**
AT4G10250	ATHSP22.0	HSP20-like chaperones superfamily protein	**675.028796**
AT1G53540	F22G10.20	HSP20-like chaperones superfamily protein	**372.497245**
AT4G34210	SK11	SKP1-like 11	**302.539198**
AT3G46230	HSP17.4	heat shock protein 17.4	**182.858664**
AT1G78720	F9K20.24	SecY protein transport family protein	**169.507199**
AT5G12030	HSP17.6A	heat shock protein 17.6A	**152.147640**
AT5G12020	HSP17.6II	17.6 kDa class II heat shock protein	**94.781906**
AT1G56410	ERD2	heat shock protein 70 (Hsp 70) family protein	**89.687999**
AT1G07400	F22G5.25	HSP20-like chaperones superfamily protein	**85.555199**
AT2G29500	F16P2.12	HSP20-like chaperones superfamily protein	**72.782097**
AT1G59860	F23H11.18	HSP20-like chaperones superfamily protein	**48.389307**
AT5G52640	HSP90.1	heat shock protein 90.1	**39.044744**
AT3G12580	HSP70	heat shock protein 70	**36.373631**
AT5G51440	MFG13.15	HSP20-like chaperones superfamily protein	**32.445293**
AT3G09350	Fes1A	Fes1A (AT3G09350.1; AT3G09350.2; AT3G09350.3)	**25.249359**
AT5G02490	T22P11.80	heat shock protein 70 (Hsp 70) family protein	**22.041901**
AT3G53230	AT3G53230	ATPase, AAA-type, CDC48 protein	**13.288580**
AT3G09440	AT3G09440	heat shock protein 70 (Hsp 70) family protein (AT3G09440.1; AT3G09440.2)	**12.352952**
AT1G16030	Hsp70b	heat shock protein 70B	**12.291764**
AT1G54050	F15I1.13	HSP20-like chaperones superfamily protein	**6.662374**
AT1G02980	CUL2	cullin 2	**5.850634**
AT5G22060	J2	DNAJ homologue 2	**5.430694**
AT5G37670	K12B20.120	HSP20-like chaperones superfamily protein	**5.174720**
AT5G56030	HSP81-2	heat shock protein 81-2 (AT5G56030.1; AT5G56030.2)	**4.741008**
AT3G60540	AT3G60540	preprotein translocase Sec, Sec61-beta subunit protein (AT3G60540.1; AT3G60540.2)	**4.264318**
AT3G08690	UBC11	ubiquitin-conjugating enzyme 11 (AT3G08690.1; AT3G08690.2)	**3.818063**
AT5G59720	HSP18.2	heat shock protein 18.2	**3.765756**
AT5G56010	HSP81-3	heat shock protein 81-3	**3.162246**
AT4G25230	RIN2	RPM1 interacting protein 2 (AT4G25230.1; AT4G25230.2)	**3.144003**
AT1G11905	AT1G11905	B-cell receptor-associated protein 31-like (AT1G11905.1; AT1G11905.2)	**3.013872**
AT3G54130	AT3G54130	Josephin family protein	**2.608099**
AT3G07370	CHIP	carboxyl terminus of HSC70-interacting protein	**2.605097**
AT1G17280	UBC34	ubiquitin-conjugating enzyme 34 (AT1G17280.1; AT1G17280.2)	**2.541532**
AT3G44110	ATJ3	DNAJ homologue 3 (AT3G44110.1; AT3G44110.2)	**2.530448**
AT3G09840	CDC48	cell division cycle 48	**2.460131**
AT5G49910	CPHSC70-2EAT SHOCK PROTEIN 70-2	chloroplast heat shock protein 70-2	**2.315013**
AT1G09080	BIP3	heat shock protein 70 (Hsp 70) family protein (AT1G09080.1; AT1G09080.2)	**2.152765**
AT1G65040	F16G16.3	RING/U-box superfamily protein (AT1G65040.1; AT1G65040.2; AT1G65040.3)	**2.147383**
AT3G48570	AT3G48570	secE/sec61-gamma protein transport protein	**2.119919**
AT5G49570	PNG1	peptide-N-glycanase 1	**2.110732**

AT5G03340	F12E4.70	ATPase, AAA-type, CDC48 protein	2.091757
AT4G24920	F13M23.60	secE/sec61-gamma protein transport protein	2.003694
AT4G21870	T8O5.80	HSP20-like chaperones superfamily protein	-3.263621
AT4G28270	RMA2	RING membrane-anchor 2	-4.194001
AT1G24320	F3I6.26	six-hairpin glycosidases superfamily protein	-7.605802
AT4G25200	HSP23.6-MITO	mitochondrion-localized small heat shock protein 23.6	40.294621
AT5G59720	HSP18.2	heat shock protein 18.2	37.984844
AT5G12030	HSP17.6A	heat shock protein 17.6A	20.449308
AT1G53540	F22G10.20	HSP20-like chaperones superfamily protein	20.151146
AT3G46230	HSP17.4	heat shock protein 17.4	20.070805
AT2G29500	F16P2.12	HSP20-like chaperones superfamily protein	17.852214
AT5G51440	MFG13.15	HSP20-like chaperones superfamily protein	15.588747
AT5G12020	HSP17.6II	17.6 kDa class II heat shock protein	14.547546
AT1G16030	Hsp70b	heat shock protein 70B	13.631347
AT1G54050	F15I1.13	HSP20-like chaperones superfamily protein	7.240733
AT1G59860	F23H11.18	HSP20-like chaperones superfamily protein	5.212793
AT1G07400	F22G5.25	HSP20-like chaperones superfamily protein	4.903909
AT5G37670	K12B20.120	HSP20-like chaperones superfamily protein	3.448514
AT5G52640	HSP90.1	heat shock protein 90.1	2.547732
AT1G18830	F6A14.8	Transducin/WD40 repeat-like superfamily protein	2.471852
AT5G51450	RIN3	RPM1 interacting protein 3 (AT5G51450.1; AT5G51450.2)	2.071171
AT3G48570	AT3G48570	secE/sec61-gamma protein transport protein	-2.027109
AT1G35620	PDIL5-2	PDI-like 5-2	-2.156311
AT1G56330	SAR1B	secretion-associated RAS 1B	-2.204647
AT2G32920	PDIL2-3	PDI-like 2-3	-2.292988
AT1G24320	F3I6.26	six-hairpin glycosidases superfamily protein	-2.417744
AT5G07340	T2I1.50	Calreticulin family protein (AT5G07340.1; AT5G07340.2)	-2.761212
AT5G50460	MBA10.8	secE/sec61-gamma protein transport protein	-2.787589
AT5G49910	CPHSC70-2EAT SHOCK PROTEIN 70-2	chloroplast heat shock protein 70-2	-2.890887
AT5G28540	BIP1	heat shock protein 70 (Hsp 70) family protein	-2.900636
AT5G56010	HSP81-3	heat shock protein 81-3	-2.924787
AT2G47470	UNE5	thioredoxin family protein (AT2G47470.1; AT2G47470.2; AT2G47470.3; AT2G47470.4)	-3.058351
AT5G42020	BIP2	heat shock protein 70 (Hsp 70) family protein (AT5G42020.1; AT5G42020.2)	-3.221884
AT4G21870	T8O5.80	HSP20-like chaperones superfamily protein	-3.467417
AT1G21750	PDIL1-1	PDI-like 1-1 (AT1G21750.1; AT1G21750.2)	-3.520060
AT1G04980	PDIL2-2	PDI-like 2-2	-3.542803
AT1G77510	PDIL1-2	PDI-like 1-2	-3.598995
AT4G28270	RMA2	RING membrane-anchor 2	-3.639839
AT3G62600	ATERDJ3B	DNAJ heat shock family protein	-3.787809
AT5G03160	P58IPK	homolog of mamallian P58IPK	-4.143879
AT5G02490	T22P11.80	heat shock protein 70 (Hsp 70) family protein	-4.392416
AT5G61790	CNX1	calnexin 1	-4.542169
AT1G09210	CRT1b	calreticulin 1b	-4.616284
AT1G56340	CRT1a	calreticulin 1a (AT1G56340.1; AT1G56340.2)	-5.071673
AT3G54960	PDIL1-3	PDI-like 1-3 (AT3G54960.1; AT3G54960.2)	-5.224140
AT4G24190	SHD	chaperone protein htpG family protein (AT4G24190.1; AT4G24190.2)	-5.337166
